# The Effect of Acute Body Unloading on Somatosensory Performance, Motor Activation, and Visuomotor Tasks

**DOI:** 10.3389/fphys.2020.00318

**Published:** 2020-04-17

**Authors:** Ashleigh Marchant, Nick Ball, Jeremy Witchalls, Gordon Waddington, Ajitkumar P. Mulavara, Jacob J. Bloomberg

**Affiliations:** ^1^Research Institute for Sport and Exercise, University of Canberra, Canberra, ACT, Australia; ^2^KBRWyle, Houston, TX, United States; ^3^NASA Johnson Space Center, Houston, TX, United States

**Keywords:** microgravity, somatosensation, active movement extent discrimination apparatus (AMEDA), visuomotor, lower limb muscle activity, proprioception

## Abstract

Evaluating countermeasures designed to reduce the impact of microgravity exposure on astronaut performance requires the development of effective methods of assessing changes to sensorimotor function in 1g analog systems. In this study, somatosensation at the ankle and fingers, lower leg muscle activity and visuomotor control were assessed using a full body loading and acute unloading model to simulate microgravity. It was hypothesized that the function of the hands and eyes are not constrained to ‘weight bearing’ postures for optimal function and would not differ between the loaded and acute unloaded conditions, whereas lower leg muscle activity and ankle somatosensation would be reduced in the acute unloaded condition. Somatosensation was recorded using the Active Movement Extent Discrimination Apparatus (AMEDA) protocol where participants were required to make an absolute judgment of joint position sense. A score closer to 1.0 demonstrates higher accuracy. Lower leg muscle activity was recorded using electromyography of major lower leg musculature to observe peak muscle activity and duration of contraction. The King Devick infrared eye tracking test was used to asses visuomotor control by monitoring saccade velocity and fixation time. In acute unloading, it was found that ankle somatosensation had decreased accuracy (loaded 0.68, unloaded 0.66, *p* = 0.045) while finger somatosensation improved (loaded 0.77, unloaded 0.79, *p* = 0.006). When acutely unloaded, peak lower leg muscle activation reduced ( > 27%) and total contraction time increased (2.02 × longer) compared to loading. Visuomotor assessment results did not vary between the loaded and acute unloaded postures, however the underlying techniques used by the participant to complete the task (saccade velocity and fixations time) did increase in acute unloaded conditions.

**Significance:**

This research provides an insight to how to the human body responds immediately to acute changes of gravitational load direction. It provides insight to the acute affects’ astronauts may encounter when in microgravity.

## Introduction

Exposure to changes in body load due to gravity alters astronauts’ gait patterns, sensorimotor coordination, and causes inconsistent control of center of mass movement patterns, such as increased anterior-posterior displacements (Lestienne and Gurfinkel, 1988; Baroni et al., 1999; Mulavara et al., 2010, 2018; Wood et al., 2015). Some changes occur immediately, while others are an adaption to longer exposure (Lestienne and Gurfinkel, 1988; Layne and Spooner, 1990). Returning astronauts often become slower and less precise in movement during the gait cycle, thereby requiring a greater amount of time to avoid obstacles (Mulavara et al., 2010). Changes in somatosensory function may be a potential cause of the maladaptation as somatosensation plays a significant role within the sensorimotor system. A combination of proprioception and tactile sensation, somatosensation provides afferent feedback to the central nervous system (CNS) about joint position and motion (Riemann and Lephart, 2002).

The impact of microgravity exposure on proprioceptive function is unclear. For example, assessing proprioception of the elbow in a simulated microgravity environment results in reduced performance of arm kinematics in upper limb tasks, while trunk inclination accuracy and ability to maintain orientation for posture is unaffected during extended exposure to microgravity (Bock, 1998; Baroni et al., 1999). Research within the Spacelab by Watt (1997) showed that astronauts’ ability to point to a target was less accurate aboard the lab in microgravity compared to Earth gravity (1g) environment. However, it was unclear whether this was because of poor limb position sense or lack of knowledge of where the target was positioned. Thus, the underlying mechanisms and degree of potential contribution from the proprioceptive system in a microgravity environment is still unknown.

Muscle spindles provide a major contribution to proprioceptive function (Ashton-Miller et al., 2001; Han et al., 2016) and changes in load stimulus with various gravitational environments may alter their operation. Assessing whether a relationship between muscle activity and somatosensory performance exists under varying load conditions, may provide insight into the mechanisms that mediate performance changes with microgravity. Microgravity studies using electromyography (EMG) of the lower limb have demonstrated that during eccentric muscle contraction, the tibialis anterior is highly activated (Kenyon and Young, 1986). Other research has shown poor co-contraction of antagonistic paired muscles, as well as excessive activity of superficial lumbosacral muscles during simulated microgravity experiments (Belavý et al., 2007; Sadeghi et al., 2016). Therefore, analyzing other aspects of the sensorimotor system in conjunction with proprioception has the potential to provide further insight to physiological changes in novel gravitational environments.

Data from the sensorimotor system normally integrates with visual information to assist the body with orientational awareness (Harris et al., 2011). In a 1g environment this is accompanied by vestibular activation providing a gravitational vertical reference. However, in a microgravity environment vestibular feedback is no longer relevant thus other systems are required to compensate. The visual system has also shown changes based on the gravitational environment. Saccadic eye movements have decreased latency and increased peak velocity relative to baseline observations when in microgravity (André-Deshays et al., 1993). As saccadic eye movement characteristics have been associated with limb proprioceptive ability, these changes could potentially contribute to poor somatosensation in a novel gravitational environment (Ren et al., 2007).

A range of methods have been used to assess somatosensory ability (Hillier et al., 2015). One such method that is considered reliable and valid is the Active Movement Extent Discrimination Apparatus (AMEDA) (Waddington and Adams, 1999). The AMEDA examines proprioception under functional conditions as a component of broader somatosensation. Participants are required to differentiate between joint positions that they have dynamically experienced, using an active self-driven movement. Despite its extensive use in a clinical and sport related setting (Waddington et al., 2000; Han et al., 2014, 2015; Lion et al., 2016; Steinberg et al., 2016), the AMEDA has not been used previously to assess somatosensory performance in microgravity modeling.

Modeling microgravity by acutely unloading the lower limbs in a supine body position provides a mechanism for reducing sensory information from the lower limbs and trunk, and if the head is kept immobile, vestibular function is minimized. Lying supine provides the opportunity to model aspects of unloading the body that occurs in microgravity without the associated costs, logistical constraints and risks of flights (Mulavara et al., 2018). Whilst in this orientation, the direction of gravitation pull is altered and no longer serves as a reliable reference to posture. This short exposure to lower limb unloading model has been used in strength training for the purpose of rehabilitation, but also balance testing in the research laboratory at the National Aeronautics and Space Administration (NASA) (Goel et al., 2017; Oddsson et al., 2007).

The primary aim of the present research was to assess ankle and finger somatosensation in an upright full weight-bearing or loaded stance and in an acute unloaded supine posture using an AMEDA approach. A secondary aim was to measure lower limb muscle activity and assess visuomotor control in the two conditions. The objective was to assess differences associated with the acute unloading, simulating the effects of immediate exposure to microgravity. It was hypothesized that ankle somatosensation scores would be reduced in the acute unloaded condition when compared to a normal loaded condition, and that the visual neuromotor and finger somatosensation abilities would not differ between the two conditions. It was predicted that muscle activity in the lower limb would be less in the acute unloaded posture. These hypotheses are based on the premise that the function of the hands and eyes are not constrained to ‘weight bearing’ postures for optimal function (Janwantanakul et al., 2003).

## Materials and Methods

Fifty-seven healthy individuals between the ages of 18 and 60 years were recruited (28 Males), nine were left hand dominant, and six were left foot dominant. Mean ± standard deviation (SD) age was 30.4 ± 1.5 years, height; 171.4 ± 1.2 cm and weight; 71.1 ± 1.9 kg. The study was completed in accordance with the University of Canberra Human Research Ethics Statement (approval number: 16-215). Participants attended the University for a single session. Written informed consent was obtained from participants prior to the commencement of the test. Exclusion criteria included any medical conditions which may affect balance and any hand or ankle injuries within the previous 3 months. Participants were asked to avoid caffeine 12 h prior to the testing and to avoid strenuous activity 24 h prior, to reduce effects of neural overstimulation or fatigue. “Baseline” Tactile Semmes-Weinstein Sensory Monofilaments (Fabrication Ent, Inc. Baseline Tactile Monofilaments, White Plains, NY, United States) were used to assess sensation within the sole of each participant’s foot to exclude reduced tactile sensation. According to the Young (2008) definition of reduced protective tactile sensation, incorrect identification of four or more sites of application of the monofilament to the sole of the foot indicates diminished sensory competency and increases the likelihood of neuropathy. The Identification of Functional Ankle Instability scale (IdFAI) was completed prior to assessment, to identify the participant’s concurrent degree of functional ankle instability (FAI) and whether this influenced performance.

### Somatosensory Test Protocol

Assessment of ankle somatosensation, finger somatosensation and visual neuromotor control was undertaken for every participant. Each assessment was completed once in upright, full weight bearing position, and once in supine, non-weight bearing position. Examination was commenced once the participant was standing, sitting or lying in the correct alignment for each test, effectively assessing immediate or acute exposure. The order in which the tests were performed was randomized using an online random number generator^1^. Previous research has determined that ankle proprioception is generally more accurate on the foot which stabilizes the body during a kicking movement (Han et al., 2013), therefore data was recorded from the ankle of the self-reported stabilizing leg. Hand dominance was determined prior to the finger proprioception assessment.

### Ankle Somatosensation

The Ankle AMEDA is a platform device comprised of a fixed, and moveable plate which swings around an axis to assess five extents of inversion ankle movement (Waddington and Adams, 1999; Waddington et al., 2000). The Ankle AMEDA measures the participant’s ability to determine discreet changes in ankle movement extent. The range of the five available inversion movements is set by adjusting the height of the stop that limits the range of the moveable platform. This creates a series of five ranges of platform rotation, relative to the horizontal, at the testing ankle. Participants were familiarized with the all five positions prior to testing. With vision occluded by looking straight ahead, participants were asked to move their ankle into inversion until a stop was reached and return to the starting, or neutral position. They were then asked to make a judgment regarding which one of five possible ankle ranges of movement had been undertaken. Each participant was tested on a series of ten repetitions of each of the five stop positions, randomly sequenced to give 50 trials.

Ankle inversion was chosen for this study for several reasons. A ceiling effect has been observed in prior plantar flexion ankle AMEDA testing (Waddington and Adams, 1999). Muscle spindles are an important contributor of proprioceptive signal for somatosensation (Han et al., 2016). Due to their smaller muscle belly size ankle inversion/eversion muscles have fewer muscle spindles than the plantar flexors. This allowed a more sensitive differentiation between participants by reducing input from bulkier musculature, and consequently provides a wider spread of somatosensory scores. This movement is also most relevant to ankle injury, since injury to the lateral aspect of the ankle joint (inversion movement) is among the most common causes of ankle instability (Hertel, 2002). Further, the idFAI was included in the study as a self-report measure of participants’ perceptions of lateral ankle instability, to determine whether ankle instability influences performance.

**FIGURE 1:**
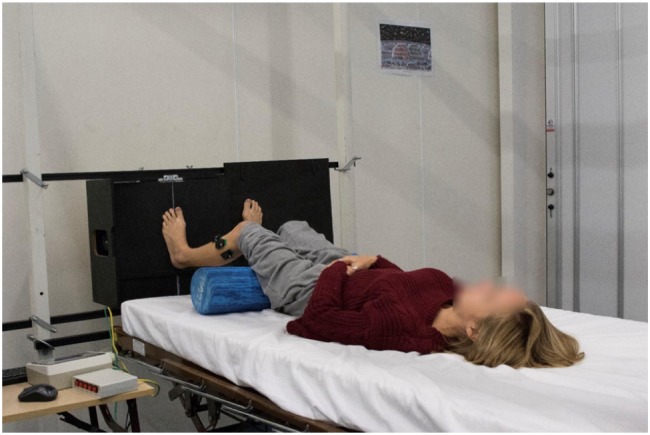
Whilst supine, the AMEDA was rotated 90° and anchored to a frame at the foot of a hospital style bed. At commencement of the assessment, hip and knee extension were maintained to replicate that of upright stance.

Two calibrated AMEDA devices were utilized for this project. When upright, participants stood up on a platform AMEDA which was resting on the floor. They stood with feet approximately hip-width apart and equal stance. Whilst supine, the AMEDA was rotated 90° and anchored to a frame at the foot of a hospital style bed (see Figure 1). Participants lay in a position comparable to upright stance. Identical hip and knee extension were maintained for both loaded and acute unloaded body positions. Minimal training is required to operate the device and predominantly controlled by movement from the subject, thus a measure of active somatosensation.

The validity and reliability of the AMEDA protocol for assessment of functional ankle proprioception has been reported previously, with a test-retest intra-class correlation coefficient of 0.80 (Waddington and Adams, 1999; Witchalls et al., 2014). To generate a somatosensory performance score from the AMEDA, nonparametric signal detection theory receiver operating characteristic analysis was used to compare responses to pairs of ankle movements (McNicol, 2005). The area under the curve (AUC) of the receiver operating characteristic (ROC) curve was used as the measurement of ankle proprioceptive sensitivity, representing participants’ ability to discriminate between the five ankle movements (Maher and Adams, 1996; Waddington et al., 2000; McNicol, 2005). Participant responses are recorded in a matrix and ROC analysis is applied to construct a score on the participants’ ability to discriminate the small changes in extent of active joint movement when moving to the different stop positions. The ROC analysis produces a number between 0.5 and 1.0, with 0.5 representing 50/50 chance of correct discrimination between stop heights and 1.0 representing perfect accuracy (see Table 1). This technique has been demonstrated as an effective tool to analyze participants responses on the AMEDA (Adams et al., 2017).

**TABLE 1:** An example of the cumulative matrix recorded for one trial on the Ankle AMEDA.

	Participant response *				
Platform stop #	*1	*2	*3	*4	*5
#1	8	1	1	0	0
#2	1	6	2	0	1
#3	2	2	1	3	2
#4	0	2	6	0	2
#5	0	0	2	2	6

*There are 5 possible stops for the platform to reach and is presented to the participant in a randomized order. The table demonstrates how many times the participant responded with each number for every presented platform stop. This participant received an AUC score 0.70.*

### Finger Somatosensation

The Finger AMEDA followed the same principles of the Ankle AMEDA, with participants being asked to differentiate between thumb and first finger pinch positions. The Finger AMEDA is a portable device comprised of two levers with five extents of movement and is used to assess proprioception of the first two digits (Han et al., 2011). With vision occluded, participants were asked to pinch their thumb and index finger until a stop was reached and then return to the starting position. Participants were familiarized with the technique prior to testing. The same unit was used for body orientations by transferring the device between chair and bed for upright and supine conditions. Both the loaded and acute unloaded body positions had identical forearm support and the wrist joint was maintained in a neutral resting flexed position. Participants had approximately 20–30 degrees of shoulder flexion when in the loaded posture as they rested their forearm in front of their trunk on the table. The elbow was maintained at full extension in the acute unloaded posture as they lay supine.

### Visual Neuromotor Control

The King Devick (KD) infrared eye tracking test was used to assess visual neuromotor control. The KD test is a measure of eye movement control, concentration, and cerebral cortex processing (Davies et al., 2012; Galetta et al., 2016), and has been shown to be sensitive to changes in visual function following concussion and sleep deprivation as well as the effects of hypoxia. The test was conducted via a laptop screen and pupil tracking device, which was positioned approximately 30-40 centimeters in front of the participant for both loaded and acute unloaded conditions.

### Electromyography

Lower limb muscle activity was recorded using EMG. Three parallel bar silver surface EMG electrodes (Delsys 8 channel Trigno wireless EMG system) were placed on three major lower leg muscles; tibialis anterior, peroneus longus, and gastrocnemius. Electrode positioning was adjacent to the mid tibial shaft (tibialis anterior), approximately 6 cm below the head of fibula (peroneus longus), and lateral head of the muscle belly approximately 5 cm distal and lateral to the popliteal fossa (lateral gastrocnemius) (Rainoldi et al., 2004). The skin was cleaned and dried prior to positioning each electrode over the corresponding muscle belly. Muscle activity was collected in the initial 90 s of each Ankle AMEDA trial, aligning with approximately ten platform rotations of the Ankle AMEDA. The electrodes remained on the participant’s leg for the entire session to ensure consistent positioning for both loaded and acute unloaded testing. Strong adhesive tape was used to ensure the electrodes remained in place (De Luca, 2002).

### Data Processing

Data from the Ankle and Finger AMEDA somatosensation test was uploaded to a Microsoft Excel spreadsheet and the AUC for discrimination between different inversion angles was calculated, as a measure of the participants’ somatosensation accuracy. The KD infrared eye tracking test provided overall completion time, number of incorrect numbers read aloud, mean fixation time and mean saccade angular velocity. Raw EMG data was analyzed using the EMGworks Analysis software package (Version 4.1, Delsys Inc, Natick). A Butterworth Notch Filter was initially applied (5–10 Hz) followed by a Second Order Butterworth Filter with Bandpass 10–400 Hz to remove artificial noise. To assess peak activity for each AMEDA movement in the 90 s period, a root-mean-square filter was applied with a window length of 50 ms. To assess the total activity used during the 90 s period, an Integration filter was applied (IEMG). Onset of muscle activity was calculated through inspection using Shewart’s protocol (Allison et al., 1993). The raw EMG values were then normalized by expressing the peak, in relation to the point of highest muscle activity across both body conditions. The subsequent points of activity were calculated as a percentage of this determined peak. Peak normalization was used based on the complexities of obtaining maximum voluntary isometric contraction from the peroneus longus. The peak normalized muscle activity for each contraction was calculated, with the mean of the peaks across all contractions used to represent muscle activity associated with each movement.

### Statistical Analysis

Statistical analyses were performed using SPSS version 23 (IBM Corp. Released 2015. IBM SPSS Statistics for Windows, Version 23.0 Armonk, NY, United States). All analyses were set with a significance level of 0.05. Comparison of outcome measures between the two body positions was determined via paired samples *t*-tests. Outcome measures analyzed were the AUC score of the Ankle and Finger AMEDA tests; overall completion time, fixation time, and saccade angular velocity of the KD infrared eye tracking test; and the mean peak normalized muscle activity, integrated muscle activity, and mean duration of muscle contraction for each condition.

To control for any effect of ankle injury history, participants were grouped into two ankle stability categories: likely FAI and unlikely FAI according to the idFAI ankle questionnaire outcome. An independent *t-*test of Ankle AMEDA scores was calculated to determine whether differences between ankle stability in individuals affects proprioceptive performance. To check for order effects an independent *t-*test was completed to compare difference between participants who were assessed in upright initially versus those who were assessed in supine initially. Participants were further grouped into six categories of order testing (i.e. ankle, finger or visual assessments). A one-way analysis of variance (ANOVA) was used to detect whether somatosensory accuracy is influenced by order effects of other areas of the body. Appendix 1 lists the order for further clarification.

## Results

Thirty of the 57 participants had a history of at least one ankle sprain in their lifetime and the IdFAI results indicated 12 individuals to have likely current FAI. There was no difference between ankle somatosensory scores for likely FAI participants and unlikely FAI participants. All participants were classed as having normal tactile sensation. The order of initial body loading posture (i.e., upright loaded or supine acute unloaded) for the tests completed did not significantly influence the Finger AMEDA, or KD infrared eye tracking test scores. However, there was a learning effect within the Ankle AMEDA where participants were more likely to perform with higher accuracy in the second Ankle AMEDA test. This learning effect was regardless of which body position was completed first. The one-way ANOVA indicated that the sequence of the different test types (i.e. order of completing ankle, finger and visual test) did not significantly affect scores (Ankle loaded *p* = 0.072; Ankle unloaded *p* = 0.202; Finger loaded *p* = 0.389; Finger unloaded *p* = 0.080, KD loaded *p* = 0.711; KD unloaded *p* = 0.926).

**FIGURE 2:**
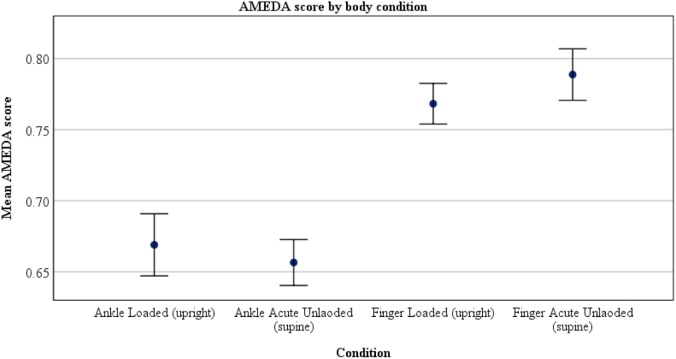
Participants were significantly less able to discriminate ankle position in the acute unloaded (supine) posture compared to the loaded (upright) posture (upright AUC score: 0.68, supine AUC score: 0.66; *p* = 0.045). The opposite was found for finger somatosensation, which improved in the supine position compared to upright (upright AUC score 0.77, supine AUC score 0.79; *p* = 0.006).

**FIGURE 3:**
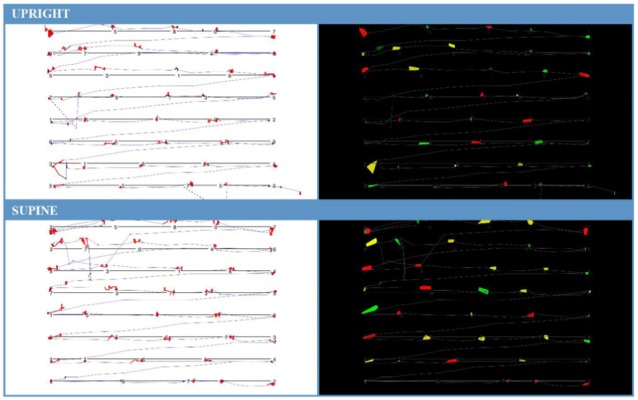
Card one of the KD infrared eye tracking test for Participant 1. Image (**A**, top) is participant in loaded or upright position. (**B**, bottom) is participant in acute unloaded or supine position. **(A)** (loaded) completion time for card one: 20.192 s, average fixation time: 367 ms, average saccade velocity: 218 deg/s. **(B)** (acute unloaded) completion time for card one: 20.229 s, average fixation time: 403 ms, saccade velocity: 215 deg/s. Increased fixation time can be seen by observing the thermal representation (right) where there is an increased number and size of colored areas in the acute unloaded orientation. This demonstrates that the participant was fixating on these points for a longer period than when upright.

Participants were significantly less able to discriminate ankle position in the acute unloaded posture compared to the loaded posture (loaded 0.68, unloaded 0.66; *p* = 0.045). The opposite was found for finger somatosensation, which improved in the acute unloaded posture compared to the loaded posture (loaded 0.77, unloaded 0.79; *p* = 0.006) (see Figure 2). The overall completion time for the KD infrared eye tracking test showed no differences between postures. Mean saccade angular velocity was greater in acute unloaded (loaded 183.44 deg/s, unloaded 198.48 deg/s; *p* < 0.001), as was fixation time (loaded 257.09 ms, unloaded 287.16 ms; *p* = 0.004). Figure 3 is a graphical representation of the KD infrared eye tracking test for participant one in loaded compared to acute unloaded.

Peak muscle activation increased by >27% during the Ankle AMEDA in the loaded condition compared to the acute unloaded. Results were statistically significant for all muscles (tibialis anterior: loaded: 54%, unloaded: 20%, *p* < 0.001; peroneus longus: loaded: 55%, unloaded: 28%, *p* < 0.001; gastrocnemius: loaded: 61% unloaded: 19%, *p* < 0.001). The total mean muscle activity (IEMG) used during each condition was significantly greater in unloaded compared to loaded for all muscles (tibialis anterior: loaded: 25%, unloaded: 43%, *p* = 0.007; peroneus longus: loaded: 24%, unloaded: 37%, *p* = 0.001; gastrocnemius loaded: 29%, unloaded: 36%, *p* = 0.040). The mean duration per muscle contraction was longer in acute unloaded (loaded: 1.37 s, unloaded: 2.78 s, *p* = 0.036) (see Table 2).

**TABLE 2:** Paired sample *t*-test loaded verses acute unloaded.

		Loaded (upright) Mean (95% CI)	Acute unloaded (supine) Mean (95% CI)	*t*	Sig
AMEDA (AUC)	Ankle	0.68(0.66,0.69)	0.66(0.64,0.67)	2.054	0.045
	Finger	0.77(0.75,0.78)	0.79(0.78,0.80)	–2.86	0.006
KD infrared eye tracking test	Completion time (s)	48.1(46.0,50.2)	48.5(45.9,51.0)	–0.708	0.482
	Saccade velocity (deg/s)	183.4(175.3,191.6)	198.5(192.4,204.63)	–4.87	< 0.001
	Fixation time (ms)	257.1(239.2,275.0)	287.2(269.1,305.2)	–3.019	0.004
Muscle contraction time (s)	Duration	1.37(1.17,1.55)	2.78(2.40,3.14)	–8.323	< 0.001
Peak activity (%)	Tibialis Anterior	54(48,59)	20(16,23)	10.364	< 0.001
	Peroneus Longus	55(50,60)	28(23,32)	8.356	< 0.001
	Gastrocnemius	61(55,67)	19(15,23)	12.983	< 0.001
IEMG (%)	Tibialis Anterior	25(20,30)	43(31,55)	–2.828	0.007
	Peroneus Longus	24(19,29)	37(32,42)	–1.854	0.001
	Gastrocnemius	29(24,34)	36(31,41)	–2.151	0.04

*Results of the Ankle AMEDA AUC score, Finger AMEDA AUC score, KD infrared eye tracking test completion time (seconds), KD infrared eye tracking test mean saccade velocity (degrees/seconds), KD infrared eye tracking test mean fixation time (milliseconds), mean muscle contraction duration (seconds), and peak muscle activity and total muscle activity (IEMG) as a percentage (%) of peak normalization for tibialis anterior, peroneus longus and gastrocnemius were compared among upright and supine. The significance (Sig) level was set at 0.05. Mean and 95% confidence interval (CI) provided for each category. Results show no significant difference between body posture for KD infrared eye tracking tests completion time. All other comparisons were significant, where ankle AMEDA AUC score was greater in loaded, finger AMEDA AUC score greater in acute unloaded, KD infrared eye tracking test mean saccade velocity and fixation time greater in acute unloaded, peak activity greater in loaded, IEMG and muscle contraction time greater in acute unloaded.*

## Discussion

This study demonstrated that mean Ankle AMEDA scores were significantly less accurate in the acute unloaded position than in the standing loaded position. In contrast, Finger AMEDA scores were significantly more accurate in the acute unloaded position than in the standing loaded position. The overall result of the KD infrared eye tracking test was not significantly different between loaded and acute unloaded positions. However, the underlying components required to achieve the composite KD score (saccade angular velocity and fixation time) did differ for the loaded position when compared to the acute unloaded position. As predicted, overall lower limb muscle activity levels reduced in the acute unloaded position compared to the loaded position.

### Ankle Somatosensation

Despite previous research identifying a relationship between ankle instability and poor AMEDA performance, the current study showed no significant difference. This has been demonstrated in one other known AMEDA study, where participants who completed a stepping AMEDA task did not vary in their performance although likely ankle instability was considered to hinder performance (Witchalls et al., 2013). In the present study, one possible factor may be the sample size of those with likely FAI, was too small. Although thirty indicated as having at least one ankle sprain in their lifetime, only twelve presented with chronic instability, according to the idFAI. The varied results show that further research should be directed in this area.

There is a general lack of research assessing ankle somatosensation in a loaded versus unloaded position using the AMEDA protocol. Furthermore, there is limited research on proprioceptive performance variability in other joints and its association with postural changes. The differences in ankle proprioceptive accuracy between the loaded and unloaded lower limb positions, as observed in this study, is hypothesized to stem from altered lower limb muscle activity and altered vestibular input. During upright stance, postural muscles of the legs and trunk are continually active and there is a level of background postural sway which provides constantly updating vestibular information about the body’s vertical alignment (Gooey et al., 2000; Madigan et al., 2006). In the unloaded posture however, standing postural muscles are less active, and provided the head is supported the vestibular mechanism is muted. Gooey et al. (2000) demonstrated this by observing that the amount of time spent above the threshold for vestibular excitable activity is limited when in supine compared to upright unipedal control task. Therefore, it is feasible that with increased sensory input from the vestibular system as well as increased postural muscle activity in the lower limb, that ankle proprioceptive acuity becomes enhanced.

Reduced ankle somatosensory performance in the acute unloading position that was found in the current study may be influenced by the degree of opportunity for movement pattern practice. As the ankle most commonly participates as a functional joint in the maintenance of loaded upright posture, proprioception is likely to be most tuned for performance in this configuration through “training.” This idea is supported by Refshauge and Fitzpatrick (1995) who compared perception of movement around the ankle in five body positions including upright and seated conditions. They found that performance was more accurate in the upright and simulated upright positions compared to the seated positions. However, the methods used by Refshauge and Fitzpatrick (1995) differed to the present study, as their participants’ head and trunk were maintained in an upright position. Because participants had constant vestibular input, this contradicts our hypothesis. Nonetheless, it supports the concept that ankle proprioception is most accurate when the body is operating in a familiar orientation. In this study there was a learning effect for the Ankle AMEDA, which has been demonstrated previously (Witchalls et al., 2014). Familiarity of a task appears to play an important role in somatosensory accuracy.

It is estimated that a trip to Mars would result in extensive losses to major human physiological systems, including the somatosensory system (Carpenter et al., 2010). For example, a six-month microgravity exposure study demonstrated an average loss of one third of gastrocnemius power, with some individuals reaching even higher levels of muscle atrophy (Fitts et al., 2010). Consequently, although the observed reduction of 0.02 on the Ankle AMEDA appears minor, it remains to be established whether this difference is *clinically* important. Recent studies have shown that a similar change in AMEDA performance, correlates to attainment of athletic level (Han et al., 2014; Dickson et al., 2019). Those in a higher class of sports performance, or hold a higher qualification in sport instructing, are significantly more accurate on the Ankle AMEDA than those in a lower class or without a qualification in sport instructing. Furthermore, research targeted at individuals with ankle instability who are at risk of injury and chronic pain have demonstrated an average of 0.03 reduction in Ankle AMEDA scores than people without ankle instability (Witchalls et al., 2012).

### Finger Somatosensation

The finding that finger somatosensory performance was enhanced when in the acute unloaded position when compared to the loaded position was unexpected. It is possible that enhanced performance in the unloaded position may be due to an increased attentional diversion to the movement task. In the acute unloaded position, vestibular feedback is minimized, and the postural muscles involved in maintaining upright stance are inactive. This reduction in total sensory input might result in less ‘noise’ for the CNS to interpret, allowing more attention on the fine motor movement task at the fingers. Research by Kramer et al. (1997) assessing proprioception using a joint position reproduction task in individuals with knee pathology, demonstrated that those with patellofemoral pain syndrome had greater accuracy of proprioception in a non-weight bearing position. The researchers argued that it may be due to an overload of information occurring when in an upright loaded stance, which supports the increase in accuracy of the Finger AMEDA in the acute unloaded posture in the present study. With increased general sensory input during an upright stance, the CNS may have to deal with increased input activity, whereas in relaxed supported supine lying (and therefore reduced sensory loading), it can provide more attention to the task. In a study assessing upper limb movement position reproduction, Papaxanthis et al. (1998) showed significantly improved upward arm movement reproduction when in microgravity compared to the 1g environment. Exposure to reduced body load conditions appears to enhance the upper limb somatosensory function as upper limb function relies much less on being in a whole body loaded posture as opposed to the lower limb.

Other researchers have reported function of the upper limb in a microgravity environment to be maintained at a level comparable to 1g. A study by Gurfinkel et al. (1993) assessed participants’ ability to draw ellipses with their arm. The task was performed in upright and supine lying in 1g and in microgravity. Results revealed no differences in elliptical drawing quality between the two upright conditions. However, Gurfinkel et al. (1993) assessed whole of upper limb movement and did not focus on localized finger movement ability. While it is evident that further research is required to understand somatosensory function of the upper limb, the results of the present study support previous upper limb motor control research, in implying that the upper limb has a greater egocentric control which may not be as heavily affected by microgravity as the exocentrically controlled lower limb.

### Vision

The KD infrared eye tracking test results demonstrated that test completion time is not significantly affected by an acute change in body position. These results compare to those of Dalecki et al. (2013) who demonstrated no change in reaction time or number of errors for a cognitive, visual based task in microgravity. Interestingly, it appears that the approach used by participants to complete the KD infrared eye tracking test of the present study differed significantly between body positions. To scan our surroundings, the visual system uses a combination of both saccadic eye movement and fixation control. In the upright loaded condition, participants demonstrated shorter fixation times with slower saccade angular velocities, whereas the opposite was true in the acute unloaded posture condition. Microgravity environments have been shown to negatively affect visual tracking and gaze hold ability, characterized by slower saccade angular velocity and reduced precision (Kornilova et al., 2012). This increased fixation time may be indicative of the participants’ unfamiliarity with performing screen-based activities in the supine position, and a resultant unanticipated cognitive load.

The current study showed that saccade velocity was slower in the upright loaded position. McColeman and Blair (2014) demonstrated that slowed saccade angular velocity correlated to increased cognitive demand, number of distractions and relevance of the task. This appears to be supported in the present study, as participants may have greater demand on the central motor control systems when in the upright loaded position. McColeman and Blair (2014) also concluded that saccade angular velocity and fixation timing are independent of one another and follow different command systems. If increased cognitive demand slows saccade angular velocity, perhaps upright loaded position could be expected to influence the KD infrared eye tracking test results in the same manner.

### Muscle Activation

The upright loaded position produced greater peak muscle activation and shorter duration of muscle activity in the lower limb compared to the supine unloaded position. In upright stance, postural muscles are continually active, also known as ‘postural tone’ (Samuel et al., 2015). Higher peak muscle activations typically occur in response to brief perturbations of postural sway that are constant when in upright standing. This mechanism is not unique to the lower limbs and occurs also in muscle activity of the trunk and neck (Jakobs et al., 1985). In supine however, less postural muscle activity is required as there is no need to overcome body weight or the constant need to adjust postural muscles with sway coordination (Jakobs et al., 1985; Samuel et al., 2015). This is relevant to the acute unloaded position in the current study, where the requirements to control sway are removed. This means that the lower limb muscles must actively position the lower limb without a passive sway component, thus generating longer contraction periods and thus greater total muscle activity. Similarly, in a microgravity environment body sway and gravitational load are not able to be used to facilitate generation of movement, so the action needs to be generated by the task specific muscles alone. We hypothesize that similar muscle activity patterns in the present study would also be evident in a microgravity environment, however further investigation in microgravity is needed.

Despite greater IEMG in supine due to the longer time under tension, the activity was occurring at a lower peak intensity than when upright. The reduced peak activation of postural muscles in supine correlates to previous microgravity research where muscle atrophy has been reported to occur among postural muscle groups (Di Prampero and Narici, 2003). Without a gravity load stimulus, muscular activity will not exceed the ‘lowest necessary level of activity’ (Edgerton et al., 2001). This therefore results in minimal or no activation of postural muscles in microgravity (or supine lying) as load is below the minimum requirement for activation. Layne and Spooner (1990) assessed anticipatory postural muscle activity in the lower limbs during rapid balance disturbances and showed the hamstrings were active in the 1g environment to maintain balance in response to arm movements. However, the muscle activity of the hamstrings was diminished once exposed to microgravity. It has been suggested that this is only true among muscles required for postural tone in 1g with the early microgravity ‘spacelab’ series of experiments, demonstrating that excessive muscle activity of phasic muscle groups occurs in the lower leg to maintain upright posture (Kenyon and Young, 1986). This was later confirmed by Edgerton et al. (2001), where astronauts’ lower leg muscle activity was observed over a 24-h period. The tibialis anterior muscles showed the most extreme change, demonstrating total muscle activity levels to be up to six times greater than when in 1g. This is consistent with the findings of the present study, indicating prolonged phasic muscle activity during the unloaded or non-weight bearing equivalent tasks. Edgerton et al. (2001) argue that this may be due to inactivity of the antagonist muscles in the microgravity environment and reduced requirement to decelerate limb movements during typical joint actions, thus enabling greater input from tibialis anterior muscles.

A possible disadvantage to interpreting the muscle activation results, is that despite significant numbers of Russian Space missions, little of these resources are referenced in the present study. This is due to a lack of relevance and application to the concepts examined in the current study. For example, European studies in collaboration with Roscosmos (Russian Space Agency) have published literature examining muscle tone among other characteristics of cosmonauts aboard the MIR missions (Zange et al., 1997; Gallasch et al., 1998). This is beyond the scope of the current project, however, it could be an area of interest for future researchers to integrate with the muscle activity patterns observed in the present study.

### Limitations

A limitation of the present study is the constraints of 1g, where gravitational pull is never altered, nor can it be without directing traveling to microgravity. Although the findings here are not directly comparable to microgravity, some mechanisms can be translated between the two environments. Through reduced head movement, vestibular function is minimized, and without muscle loadings associated with postural control, muscle activity is minimized, thus mimicking changes occurring in microgravity (Pavy-Le Traon et al., 2007). This type of study design has been used in previous NASA human research models and in early rehabilitation following stroke (Oddsson et al., 2007, 2015; Goel et al., 2017).

Another limitation is the varied elbow flexion angle between upright loaded and supine unloaded positions. As the angle was altered, it is unknown whether this had any effects on somatosensation. Theoretically, there should be no effect when supine, as forearm flexors were in a stretched position, thus reducing the strength of mechanoreceptors, specifically muscle spindles which have a large role on proprioception and are thought to be more effective when contracted (Ashton-Miller et al., 2001). However, this is speculative and further examination is required. Should the tests be repeated, it would be recommended that the Finger AMEDA be rotated to a platform, like the modified Ankle AMEDA.

## Conclusion

The findings of the present study demonstrate that somatosensory acuity of the ankle is reduced when participants are in a supine (unloaded) position, compared to an upright standing (loaded) position, but somatosensory acuity seems to be improved in the fingers. While the eye tracking test completion time did not show overall differences between body orientations, it did reveal different strategies used by participants to complete the test in the different positions.

As it is the first in its design, the current study holds potential to investigate the topic further. It is theorized that greater duration spent in reduced loading will continue to have a negative impact and ongoing decline to results on the Ankle AMEDA as a representation of proprioception. In bed rest studies, the human body accommodates to the lack of gravitational resistance, much in the same manner as microgravity (Pavy-Le Traon et al., 2007). With time human systems deteriorate, and it is that deterioration that is hypothesized to be seen in the AMEDA should prolonged exposure occur.

Future research should explore the underlying mechanisms behind these transient alterations to somatosensory acuity during positional changes, for example whether the changes in somatosensory performance vary consistently with the degree of loading and whether these changes are consistent with changes seen with prolonged exposure. Future research should also examine methods of applying appropriate prevention or in-flight training protocols for astronauts exposed to microgravity to reduce the deleterious effects of somatosensory performance loss. The aim being to reduce the impact of microgravity exposure, thereby increasing safety and performance of crew members.

## Data Availability Statement

The datasets generated for this study are available on request to the corresponding author.

## Ethics Statement

The studies involving human participants were reviewed and approved by the University of Canberra Human Research Ethics Statement (approval number: 16-215). The patients/participants provided their written informed consent to participate in this study. Written informed consent was obtained from the individual(s) for the publication of any potentially identifiable images or data included in this article.

## Author Contributions

AM conducted the experiments, acquired and analyzed the data, and drafted the manuscript. GW, APM, and JB conceptualized and created the study deign. NB and JW assisted in the experimental design. NB, JW, and GW aided in interpreting the results and in drafting the manuscript. APM and JB aided in revising the manuscript. All authors contributed to the approval of the version of the manuscript to be published.

## Conflict of Interest

APM was employed by the company KBRWyle. The remaining authors declare that the research was conducted in the absence of any commercial or financial relationships that could be construed as a potential conflict of interest.

## Footnotes

1 https://www.randomizer.org/

## Appendix

### Order of Somatosensation Testing Categories

**Table:** Participants were randomly organized into one category from the possible six combinations.

One	Ankle AMEDA, Finger AMEDA, KD test
Two	Ankle AMEDA, KD test, Finger AMEDA
Three	Finger AMEDA, KD test, Ankle AMEDA
Four	Finger AMEDA, Ankle AMEDA, KD test
Five	KD test, Ankle AMEDA, Finger AMEDA
Six	KD test, Finger AMEDA, Ankle AMEDA

## Abbreviations

1g: Earth’s surface gravity (9.81m/s^2^)
AMEDA: Active Movement Extent Discrimination Assessment
AUC: area under the curve
CI: Confidence Interval
CNS: central nervous system
EMG: electromyography
FAI: functional ankle instability
IdFAI: identification of functional ankle instability
IEMG: integrated activity (total muscle activity)
KD: King Devick
SD: standard deviation.
